# Under the Christmas Tree: Belowground Bacterial Associations With *Abies nordmanniana* Across Production Systems and Plant Development

**DOI:** 10.3389/fmicb.2020.00198

**Published:** 2020-03-04

**Authors:** Adriana M. Garcia-Lemos, Alex Gobbi, Mette Haubjerg Nicolaisen, Lars H. Hansen, Thomas Roitsch, Bjarke Veierskov, Ole Nybroe

**Affiliations:** ^1^Department of Plant and Environmental Sciences, Faculty of Science, University of Copenhagen, Frederiksberg, Denmark; ^2^Department of Adaptive Biotechnologies, Global Change Research Institute, CAS, Brno, Czechia

**Keywords:** *A. nordmanniana*, Christmas trees, rhizosphere, beneficial bacteria, microbiome, nitrogen cycling, nitrogen-fixing bacteria, denitrifying bacteria

## Abstract

*Abies nordmanniana* is an economically important tree crop widely used for Christmas tree production. After initial growth in nurseries, seedlings are transplanted to the field. Rhizosphere bacterial communities generally impact the growth and health of the host plant. However, the dynamics of these communities during *A. nordmanniana* growth in nurseries, and during transplanting, has not previously been addressed. By a 16S rRNA gene amplicon sequencing approach, we characterized the composition and dynamics of bacterial communities in the rhizosphere during early plant growth in field and greenhouse nurseries and for plants transplanted from the greenhouse to the field. Moreover, the N-cycling potential of rhizosphere bacteria across plant age was addressed in both nurseries. Overall, a rhizosphere core microbiome of *A. nordmanniana*, comprising 19.9% of the taxa at genus level, was maintained across plant age, nursery production systems, and even during the transplantation of plants from the greenhouse to the field. The core microbiome included the bacterial genera *Bradyrhizobium*, *Burkholderia*, *Flavobacterium*, *Pseudomonas*, *Rhizobium*, *Rhodanobacter*, and *Sphingomonas*, which harbor several N-fixing and plant growth–promoting taxa. Nevertheless, both plant age and production system caused significant changes in the rhizosphere bacterial communities. Concerning community composition, the relative abundance of Rhizobiales (genera *Rhizobium*, *Bradyrhizobium*, and *Devosia*) was higher in the rhizosphere of field-grown *A. nordmanniana*, whereas the relative abundance of Enterobacteriales and Pseudomonadales (genus *Pseudomonas*) was higher in the greenhouse. Analysis of community dynamics across plant age showed that in the field nursery, the most abundant bacterial orders showed more dynamic changes in their relative abundance in the rhizosphere than in the bulk soil. In the greenhouse, age-dependent dynamics even occurred but affected different taxa than for the field-grown plants. The N-cycling potential of rhizosphere bacterial communities showed an increase of the relative abundance of genes involved in nitrogen fixation and denitrification by plant age. Similarly, the relative abundance of reported nitrogen-fixing or denitrifying bacteria increased by plant age. However, different community structures seemed to lead to an increased potential for nitrogen fixation and denitrification in the field versus greenhouse nurseries.

## Introduction

The development of complex and intimate relationships between plants and their associated microbiota is crucial for plant growth, health, and productivity (Braga et al., 2016; Rosenberg and Zilber-Rosenberg, 2016; Niehaus et al., 2019), and the rhizosphere represents a hotspot for these interactions (Philippot et al., 2013). Beneficial rhizosphere microorganisms can improve plant growth and nutrient acquisition by their production of phytohormones (Bais et al., 2006; Chaparro et al., 2014; Zhalnina et al., 2018) and their involvement in nutrient cycling (Mendes et al., 2014). Furthermore, beneficial microorganisms may antagonize plant pathogens and induce plant resistance to biotic (plant pathogenic nematodes, insects, fungi, and bacteria) or abiotic (salinity, drought, and heat) stressors (Lau and Lennon, 2012; Shi et al., 2014; Hardoim et al., 2015; Santhanam et al., 2015; Chu et al., 2016; Plett and Martin, 2018).

The composition of the rhizosphere microbial communities has previously been particularly well studied for annual plants, including several important crop species (Caliz et al., 2015; Schlemper et al., 2017). These studies have revealed the importance of soil characteristics, management, and plant developmental stage on the assembly of the rhizosphere microbiome. Fewer studies have addressed the rhizosphere microbiota in perennial plants, where each growing season represents a small part of the plant’s lifetime, and in particular, the bacterial communities are understudied (Wagner et al., 2016; Haas et al., 2018; Li et al., 2018). Perennial tree crops, including several conifers, are a source of products of economic importance, for example, wood, paper, and Christmas trees. The tree crops are furthermore responsible for important ecosystem services such as carbon sequestration (Mercado-Blanco et al., 2018). Conifer tree crops are abundant in the Boreal regions, where low soil nitrogen (N) often limits plant growth (Haas et al., 2018). This limitation makes plants more dependent on interactions with microorganisms involved in the provision of N such as N-fixing bacteria and ectomycorrhizal (EM) fungi (Mercado-Blanco et al., 2018).

The conifer Nordmann fir *Abies nordmanniana* (Stev.) Spach is widely used as a Christmas tree in Europe, where more than 30 million trees are produced annually (Bräuner Nielsen et al., 2011). The plants are usually reared in field or greenhouse nurseries. When the plants are 2–4 years old, they are removed from the nursery and transplanted for production in the forest or plantation.^1^ Tree growth is often stunned for a few years after transplantation before optimal growth is regained, and the delayed growth causes an important economic loss. Rhizosphere beneficial microorganisms could have a potential to enhance the growth and health of this important Christmas tree species. Consequently, the establishment and maintenance of beneficial rhizosphere microbial communities during early growth stages, and during transplanting, might be a prerequisite for the development of more efficient and sustainable production systems.

At present, little is known about the *A. nordmanniana* rhizosphere microbiota. However, a recent study on 3-year-old plants documented the ability of *A. nordmanniana* to select specific rhizosphere microbial communities from field soils and identified several potentially beneficial bacterial taxa as part of the core rhizosphere microbiome (Garcia-Lemos et al., 2019). Furthermore, differences in the fungal communities associated with growth-retarded versus normally growing plants indicated a dominance of potential pathogens in the growth-retarded plants (Garcia-Lemos et al., 2019). However, the effects of different soil types and production systems (field vs. greenhouse nursery) on rhizosphere bacterial communities have not so far been addressed. Moreover, the dynamics of these communities during early plant development and during transplanting in the field remains unknown.

For annual crop plants, it is well established that soil characteristics and management affect the establishment of beneficial plant–microbe associations (Kuramae et al., 2012; Chaparro et al., 2014; Wang et al., 2017). For example, the soil physicochemical conditions and management practices can shape both the rhizosphere and the bulk soil bacterial communities, and individual factors such as pH, moisture, and nutrient content are important drivers (Marschner et al., 2004; Cassman et al., 2016; Anderson et al., 2017). Plant development is another important factor shaping the rhizosphere microbial communities of annual plants under both field and greenhouse conditions (Houlden et al., 2008; Micallef et al., 2009; Chaparro et al., 2014; Marques et al., 2014; Na et al., 2017, 2019). The changes in rhizosphere microbial community composition with annual plant development stage are probably due to changed composition of root exudates, which can also influence the N-cycling microorganisms in the rhizosphere (Henry et al., 2008; Li et al., 2017) and alter the relative abundance of N-cycling genes (Hai et al., 2009). For long-lived host plants as the tree crops, more stable interactions may be established with their associated microbiota (Mercado-Blanco et al., 2018). Hence, it could be hypothesized that the structure of the rhizosphere communities in perennial crops is primarily shaped by persistent changes, for example, in environmental conditions and less by plant development.

In this study, we focus on the effects of plant age, nursery management, and replanting on the root- associated bacterial communities of *A. nordmanniana*. We address the following questions: (1) Do the composition and N-cycling potential of rhizosphere bacterial communities change over 3 years of plant growth at the nursery stage? (2) How much does the interaction between plant development and production system affect these communities? (3) Can the *A. nordmanniana* plants maintain a rhizosphere core microbiome across all nursery growing stages? And (4) are the bacterial communities stable during transplantation of the plants from greenhouse nursery conditions to the field?

## Materials and Methods

### Sampling Site and Plant Material

The current study was conducted in Denmark at two Norman fir (*A. nordmanniana*) nurseries with different production systems. Primo Plant Ejendomme ApS, located in Hadsund (56°44′22.7′′ N, 10°03′36.7′′ E), is a field nursery where plants at different developmental stages (from seedlings to 5-year-old plants) are grown in separate field stands. The nursery Himmerlands ApS, located in Storvorde (56°56′06.7′′ N, 10°06′27.4′′ E), is a greenhouse nursery where *A. nordmanniana* plants are grown in Jiffy^®^ pots containing a peat-based growth medium for 2 years, before seedlings are transplanted to production beds in the field.^2^ *A. nordmanniana* production at both nurseries derived from the same parental seed source: Berritzgaard F 665, which has been selected for uniformity in traits of importance for Norman fir production for the Christmas tree market since 1991 (providers Levinsen and Abies A/S, Gørløse, Denmark).

### Plant and Soil Collection

One, 2-, and 3-year-old *A. nordmanniana* plants were sampled at the field nursery, whereas only 1- and 2-year-old plants were sampled at the greenhouse nursery because of the earlier transplanting from the Jiffy^®^ pot production system. At each sampling site, five plants of *A. nordmanniana* (biological replicates) per age were collected. The collections were made between July and August 2017, when possible (after agreements with the Christmas tree growers for sampling facilities). At the field nursery, plants with surrounding bulk soil were sampled using a shovel on July 13, 2017. At the greenhouse nursery, entire Jiffy^®^ pots were sampled on August 21, 2017. Additionally, one bulk soil sample or growth medium sample for each plant age was obtained. Each sample was composed of three subsamples that were randomly collected and subsequently pooled into a 500-g sample. The plants and the soil samples were placed individually in clean plastic bags and transported to the Department of Plant and Environmental Sciences at the University of Copenhagen, Frederiksberg, Denmark, where they were kept at 4°C for 11/2 days before analysis.

Eurofins Agro Testing Denmark^3^ performed the soil and growth medium composition measurements with ISO-certified procedures. Method specifications can be found at www.eurofins.dk. The soil analyses included the quantification of macronutrients and micronutrients, soil organic matter and soil texture, and soil pH. For molecular analyses of plant samples, loosely attached soil was initially removed from the full root systems with a clean brush. Subsequently, the root samples including primary and secondary roots (∼3- to 10-mm diameter and ∼12- to 20-cm length) were processed together with the soil firmly adhering to the roots. Samples were kept at −20°C until further analyses.

### Transplantation Experiment

To establish the relative role of soil type and nursery management (field vs. greenhouse) on the *A. nordmanniana* bacterial rhizosphere community, a transplantation experiment between the two sampling sites was performed. Two-year-old plants from the greenhouse nursery (Himmerlands ApS) were transplanted to the field nursery stand of 2-year-old *A. nordmanniana* plants (Primo Plant Ejendomme ApS). Plants were carefully removed from the Jiffy^®^ pot, and loosely attached soil was removed by hand shaking before planting in the field. The transplantation experiment was initiated in 2017, and plants were collected at T0 = initial sampling of plants from the greenhouse nursery before transplantation, T1 = transplanted plants collected 3 months after transplantation, T2 = transplanted plants collected 6 months after transplantation, and T3 = transplanted plants collected the 9 months after transplantation. At each sampling time, five plants were collected and considered as biological replicates. For the transplantation experiment, the same procedures as mentioned above for sample preparation and transport were applied.

### DNA Extraction and Amplicon Sequencing of the 16 rRNA Gene

DNA was extracted from entire root systems with firmly adhering soil. The frozen roots that were kept at −20°C were cut into short fragments using a sterile scalpel. They were subsequently macerated in a mortar with liquid N to obtain finely ground samples representing plant material and root associated soil (referred to as rhizosphere samples hereafter). DNA was extracted from 0.25 g of rhizosphere or bulk soil samples using the PowerSoil^®^ DNA Isolation Kit (Mo Bio Laboratories, Carlsbad, CA, United States), following the manufacturer’s extraction protocol. After DNA extraction, DNA concentration and purity were assessed using a NanoDrop 2000c spectrophotometer (Thermo Scientific, Wilmington, DE, United States) (Desjardins and Conklin, 2010). DNA samples were sent for amplicon sequencing at Macrogen Inc. (Seoul, South Korea). Amplicons were obtained from the variable region V3–V4 of the 16S rRNA gene using primer pair Bakt_341F (CCTACGGGNGGCWGCAG) and Bakt_805R (GACTACHVGGGTATCTAATCC) (Roos et al., 2013) and sequenced using the Illumina MiSeq platform (paired end, 2 × 301 bp).

### Quantification of Functional Genes Involved in N Cycling by Quantitative Polymerase Chain Reaction

The copy numbers of genes involved in N cycling (*nirK*, *nir*S, *nosZ*, *nifH*, and *amoA*), as well as of the 16S rRNA gene, were quantified using quantitative polymerase chain reaction (qPCR). The qPCR was performed using an Mx3000P^®^ qPCR system (Agilent Technologies, Santa Clara, CA, United States). Briefly, 20-μL reaction mixtures included 10 μL of the Brilliant III Ultra-Fast SYBR^®^ Green Low ROX qPCR Master Mix (Agilent Technologies), 0.015 μM of BSA (Thermo Fisher Scientific, Waltham, MA, United States), 0.4 μM of each primer (Supplementary Table S1), and 2 μL of template DNA (1–10 ng/μL). The qPCR thermal cycling consisted of an initial cycle of 95°C for 3 min, followed by 40 cycles of 95°C for 20 s, annealing temperature (Supplementary Table S1) for 30 s, and a final extension for 1 min at 95°C.

Standard curves were constructed for the 16S rRNA gene and the N-cycling genes based on fragments amplified from bulk soil samples using specific primers (Supplementary Table S1). Initially, 50-μL PCR reaction mixtures with final concentrations of 3 μM MgCl_2_, 0.5 μM dNTP, 0.2 μM of reverse and forward primers, 0.05 U/μL Taq DNA polymerase (Sigma-Aldrich, St. Louis, MO, United States), 1 × PCR buffer without MgCl_2_, and 2 μL of DNA template were run at 95°C for 5 min, followed by 35 cycles of 95°C for 30 s, annealing temperature (Supplementary Table S1) for 30 s, 72°C for 30 s, and a final extension at 72°C for 1 min.

Next, the PCR amplicons were purified using the QIAquick Gel Extraction Kit (Qiagen, Santa Clara, CA, United States) and cloned into competent TOP10 *Escherichia coli* cells using the TOPO^®^ TA Cloning^®^ Kit (Invitrogen, Carlsbad, CA, United States) with the pCR^TM^2.1-TOPO^®^ vector according to the manufacturer’s instructions. These standard plasmids carrying insertions of target genes were purified and sequenced (GATC Biotech, Ebersberg, Germany) in order to verify the identity of the inserts. Tenfold dilution series were performed for each standard curve. Standard curves spanned a dynamic range from 1.67 × 10^10^ to 2.39 × 10^10^ copies/μL.

### Data Analysis

Sequencing data were analyzed using QIIME 2 v. 2018.11 (Bolyen et al., 2019) following the pipeline described previously (Gobbi et al., 2019). Briefly, demultiplexed reads from the Illumina MiSeq were trimmed and truncated to remove primers and adapters as well as bases with low quality. Thereafter, the reads were denoised, chimera checked, and dereplicated using a DADA2 denoise-single plugin (Callahan et al., 2016). The output was rarefied to 33,000 reads per sample using the Qiime feature-table rarefy, after filtering sequences belonging to chloroplast and mitochondria. Then, a multiple-sequence alignment was performed using MAFFT (Katoh and Standley, 2013). Based on the alignment, a phylogenetic tree was generated using FastTree (Price et al., 2010). The 16S rRNA gene amplicon sequences were deposited in NCBI’s Sequence Read Archive under BioProject PRJNA515250.

Alpha and beta diversity analyses of the bacterial communities were completed using the q2-diversity plugin (Lozupone and Knight, 2005). The core-metrics-phylogenetic method based on the rarefied sequence-variant table was used to perform the analysis. For the alpha diversity, phylogenetic diversity was measured based on Faith-pd score (Faith, 1992) and visualized through boxplots and rarefaction curves. Differences in alpha diversity were statistically evaluated using the Kruskal–Wallis test [a non-parametric method substituting analysis of variance (ANOVA) when the normal distribution of data cannot be assumed], and the resulting *P*-values of the alpha diversity comparisons were based on the medians of phylogenetic diversity calculated between the different analyzed samples. *P*- and *q*-values < 0.05 were considered significant. For beta diversity, the dissimilarity of the bacterial communities was calculated using principal coordinate analysis (PCoA), which was generated using a distance matrix based on the unweighted UniFrac distances (Lozupone and Knight, 2005), and the PCoA plots were visualized through Emperor (Vázquez-Baeza et al., 2013).

A permutational multivariate ANOVA (PERMANOVA) based on the unweighted UniFrac distances with 999 permutations was used to access the statistical significance of the bacterial community differences. All these statistical tests were performed using QIIME2 v2018.11. Additionally, taxonomic assignments were done through qiime feature-classifier classify-sklearn, in which a pretrained Naïve-Bayes classifier with Greengenes v_13.8^4^ was used. Taxa bar plots were built using the plugin qiime taxa bar plot. The construction of heatmaps of amplicon sequence variant (ASV) relative abundances at order level were made using the R package “ampvis2” v. 2.4.6 (Andersen et al., 2018). The differences in taxa abundance at the phylum and genus level (between the rhizosphere and bulk soil samples at both sampling sites and between plant ages) were compared using Mann–Whitney *U* test using PAST version (2.17).

Venn diagrams were constructed using the software Venny (Oliveros, 2007), to determine the rhizosphere core microbiome in the rhizosphere across sampling site and plant age. For this, the filtered list of taxa in the rhizosphere samples at the genus level was used. For the constructed Venn diagram, the mean relative abundance of the shared taxa at the genus level was used to construct stacked bar plots. Finally, for the qPCR data analysis, the absolute gene copy numbers were calculated based on each standard curve. The relative abundance of the N-cycling genes was calculated after normalization with the 16S rRNA gene copy numbers. The significance of differences of the relative abundances of functional genes was determined by one-way ANOVA, and the relative abundance of each gene among samples was compared using Student *t*-test using PAST version (2.17). *P*-values < 0.05 were considered significant.

## Results

### Soil Characteristics

Bulk soil samples from the field-nursery were classified as sandy loams with 5–7% clay and 84–90% sand. Concentrations of the measured elements, the organic matter content, and the soil pH showed only small differences between stands of 1-, 2-, or 3-year-old plants. However, slightly higher amounts of manganese, phosphorus, and iron were documented for bulk soil in the area with 3-year-old plants (Supplementary Table S2).

Jiffy^®^ pots used in the greenhouse production system contained approximately 97% of Canadian Sphagnum Peat Moss according to information provided^5^ by Jiffy Products Ltd. This peat-based growth medium contained a considerably higher amount of organic matter (>30-fold) and had higher contents of N, manganese, boron, iron, and zinc than the field nursery soil (Supplementary Table S3). In contrast, the pH was one unit lower compared with the field nursery soil. The composition of the growth medium did not vary between Jiffy^®^ pots containing 1- and 2-year-old plants (Supplementary Table S3).

### Diversity of Bacterial Communities

From the dataset used for bacterial community analysis (*n* = 50 samples), a total of 20,114 ASVs were obtained. These ASVs appeared a total of 3,272,443 times (frequency) among all samples, which corresponds to the total number of reads after quality filtering and trimming. In the rarefaction curves of the bulk soil, peat-based growth medium, and rhizosphere samples, a plateau was observed at a sequencing depth of 15,000 reads, indicating that the sampling size and effort were sufficient to cover the complete diversity of the bacterial communities in the current environments (Supplementary Figure S1).

**FIGURE 1:**
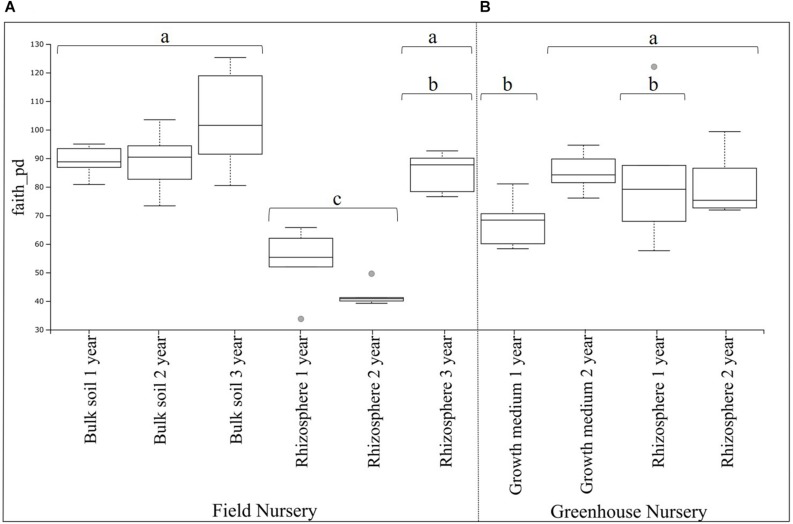
Boxplot of belowground bacterial alpha diversity of *Abies nordmanniana*. **(A)** Bacterial alpha diversity in bulk soil and rhizosphere samples by plant age at the field nursery. **(B)** Bacteria alpha diversity in the peat-based growth medium and rhizosphere samples by plant age at the greenhouse nursery. Alpha diversity was measured by Faith’s phylogenetic diversity index (faith_pd). Different letters represent significant differences with the corrected *P*-value (*q*-value < 0.05), after Kruskal–Wallis pairwise comparisons, and overlapping letters mean non-significant differences. Gray dots represent samples categorized as outliers, and each error bar represents the SD, *n* = 5.

The phylogenetic diversity analysis of the field nursery samples showed a higher (*P* < 0.01) phylogenetic diversity of the bulk soil bacterial communities as compared with the rhizosphere bacterial communities (Figure 1A). For the rhizosphere samples, a higher (*P* < 0.01) phylogenetic diversity was found in the 3-year-old plants, when compared with the rhizosphere samples from 1- and 2-year-old plants (significant differences shown by the different letters in Figure 1A).

For the greenhouse samples, the phylogenetic diversity of bacterial communities in the peat-based growth medium was higher (*P* < 0.01) for 2-year-old plants, compared with the 1-year-old plants (significant differences shown by the different letters in Figure 1B). In contrast, there were no significant differences in the phylogenetic diversity of rhizosphere bacterial communities by plant age (Figure 1B). Interestingly, the phylogenetic diversity of the rhizosphere bacterial communities was not different from that of bacterial communities in the peat-based growth medium. Individual *P*- and corrected *q*-values after the Kruskal–Wallis pairwise comparisons for both field and greenhouse samples are found in Supplementary Table S4.

**FIGURE 2:**
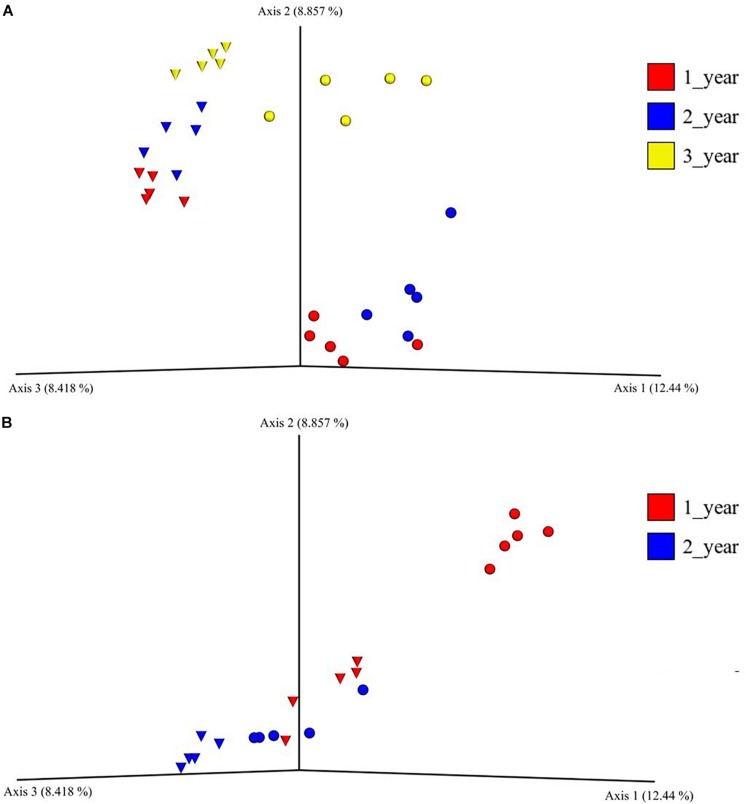
Principal coordinate analysis plot of bacterial bulk soil communities and of bacterial rhizosphere communities from 1-, 2- and 3-year-old (field nursery only) *A. nordmanniana* plants. Samples were collected in a field nursery **(A)** or a greenhouse nursery **(B)**. Analysis is based on unweighted-UNIFRAC distances. Inverted triangles represent bulk soil samples in the field nursery and peat-based growth medium samples in the greenhouse nursery. Circles represent rhizosphere samples at both sampling sites.

The beta diversity of the bacterial communities in the field nurseries was measured by unweighted UniFrac distances and visualized using PCoA. The analysis showed that the bacterial communities clustered according to plant age and that bulk soil and rhizosphere communities clustered apart (Figure 2A). Hence, the rhizosphere and bulk soil bacterial communities in the field nursery were dissimilar and even separated by plant age (Figure 2A). This was confirmed by PERMANOVA, which indicated a significant difference between treatments (sampling site, plant age, bulk soil, growth medium, and rhizosphere) (*P* < 0.001). Individual *P*-values after the pairwise PERMANOVA are found in Supplementary Table S5.

In the beta diversity analysis of the greenhouse nursery, a clear separation of the bacterial communities in the rhizosphere and in the peat-based growth medium was only observed for the 1-year-old plants (Figure 2B). Surprisingly, the rhizosphere bacterial communities of 2-year-old plants clustered together with the bacterial communities in the peat-based growth medium of the 1-year-old plants (Figure 2B). Moreover, a PERMANOVA indicated a significant difference across all treatments (*P* < 0.001), and individual *P*-values after the pairwise PERMANOVA are found in Supplementary Table S5.

### Composition of Bacterial Communities in Bulk Soil, Peat-Based Growth Medium, and Rhizosphere

**FIGURE 3:**
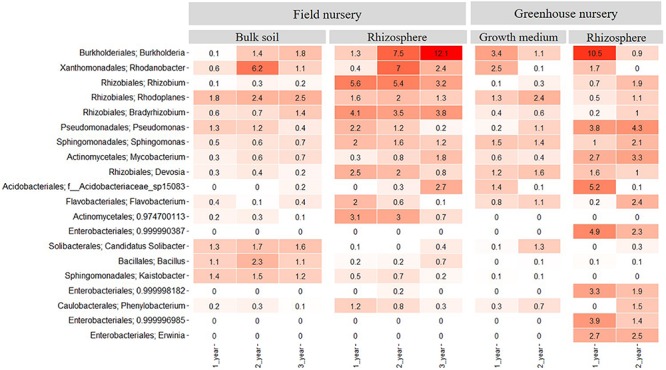
Heatmap showing the relative abundance of the 20 most abundant bacterial taxa at genus level across all samples. Samples come from bulk soil (field nursery), growth medium (greenhouse nursery), and from rhizosphere samples of 1-, 2-, and 3-year-old *A. nordmanniana* plants from either the field or the greenhouse nursery. Numbers represent the relative abundance percentages, and the darker shades of red in the figure represent the higher percentages of relative abundance at the bacterial genus level, whereas the lighter colors represent the lowest relative abundance at the bacterial genus level per sample.

In the field nursery, 46 bacterial phyla were identified when considering both rhizosphere and bulk soil samples and all plant ages. In the bulk soil, Proteobacteria was more abundant than any other phylum (*P* < 0.05) with relative abundances of 30–50% across samples (Supplementary Figure S2). Actinobacteria and Acidobacteria were also abundant in the bulk soil, with relative abundances of 10–20%. In the rhizosphere samples, Proteobacteria was more abundant than any other phylum (*P* < 0.05), with relative abundances of 50–65%. Actinobacteria and Bacteroidetes were also abundant with relative abundances between 5 and 15% (Supplementary Figure S2). At the plant age level, the relative abundance of *Burkholderia* increased significantly with plant age (*P* < 0.05) and reached a relative abundance of 12% in the 3-year-old plants. In contrast, *Rhizobium*, *Devosia*, and *Pseudomonas* showed higher relative abundances in the 1- and 2-year-old plants compared with the 3-year-old plants (Figure 3). The relative abundance of *Rhodanobacter* was different as it peaked (*P* < 0.05) in the 2-year-old plants.

In the greenhouse nursery, 36 bacterial phyla were identified across the rhizosphere samples and the peat-based growth medium samples including all plant ages (Supplementary Figure S3). In the peat-based growth medium samples, Proteobacteria was more abundant than any other phylum (*P* < 0.05) as even observed for the field bulk soil samples. However, Bacteroidetes was more abundant in the peat-based growth medium samples than observed for the bulk soil samples in the field, whereas the opposite was observed for Acidobacteria and Actinobacteria. For Acidobacteria, several genera included in subdivision 1; that is, *Terriglobus*, *Granulicella*, *Edaphobacter*, and *Koribacter* were found in higher relative abundance the field bulk soil samples compared with the greenhouse peat-based growth medium (data not shown). Nevertheless, the relative abundance of these genera was lower than the 20 most abundant bacterial taxa at the genus level shown in Figure 3 for both sampling sites. At the genus level, *Burkholderia* and *Rhodanobacter* were more abundant (*P* < 0.05) in the peat-based growth medium of the 1-year-old plants (Figure 3), whereas *Rhodoplanes*, *Pseudomonas*, and *Solibacter* were more abundant (*P* < 0.05) in the peat-based growth medium of 2-year-old plants (Figure 3).

In the rhizosphere of the greenhouse samples, Proteobacteria was more abundant than any other phylum (*P* < 0.01), with relative abundances of 60–78%. Additionally, Actinobacteria showed relative abundances of 10–17% (Supplementary Figure S3). Several genera showed dynamics with plant age. For example, *Burkholderia*, as well as different taxa from the order Enterobacteriales, had higher relative abundance (*P* < 0.05) in the rhizosphere of 1-year-old plants as compared to 2-year-old plants (Figure 3). In contrast, higher relative abundances of *Pseudomonas*, *Mycobacterium*, and *Flavobacterium* were observed in the rhizosphere of 2-year-old plants (Figure 3).

When comparing all samples across field and greenhouse nurseries, the genera with highest relative abundances were *Burkholderia*, *Rhodanobacter*, *Rhizobium*, *Rhodoplanes*, *Bradyrhizobium*, *Pseudomonas*, *Sphingomonas*, *Mycobacterium*, and *Devosia*. These genera were more abundant (*P* < 0.05) than any other genus and frequently enriched in the rhizosphere (Figure 3). When comparing the rhizosphere samples from greenhouse and field nurseries, Rhizobiales (*Rhizobium*, *Bradyrhizobium*, and *Devosia*) presented a higher (*P* < 0.05) relative abundance in the rhizosphere samples from the field nursery than observed for the rhizosphere samples in the greenhouse nursery, whereas the opposite was observed for *Pseudomonas* and different genera of Enterobacteriales.

### *A. nordmanniana* Rhizosphere Core Microbiome Across Field and Greenhouse Nurseries

**FIGURE 4:**
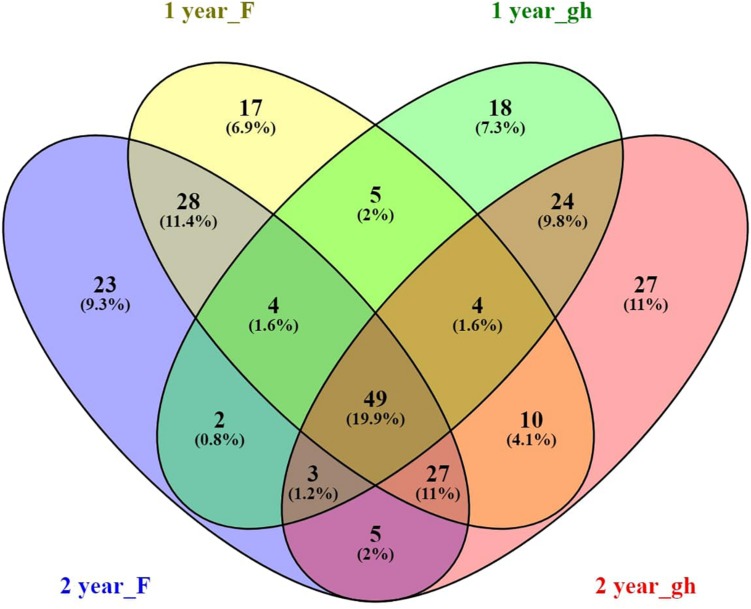
Venn diagram of the *A. nordmanniana* core rhizosphere bacterial community, analyzed at the genus level. *A. nordmanniana* plants (1 and 2 years old) were collected at field (F) and greenhouse (gh) nurseries in Denmark. Numbers represent the amount of shared and unique taxa for each plant age and site, and the numbers in parenthesis show the percentage of contribution of the core taxa in relation to the total number of taxa at genus level identified. “year” refers to the plant age (1 or 2 years old).

In order to find the taxa that have an affinity for the rhizosphere of *A. nordmanniana* across the different nursery production systems (field and greenhouse) and plant ages, we identified the core microbiome in the rhizosphere of 1- and 2-year-old plants from both sampling sites. The *A. nordmanniana* bacterial core microbiome constituted 19.9% of the identified taxa at the genus level (Figure 4). The core microbiome was constituted by taxa representing *Burkholderia*, *Rhizobium*, *Bradyrhizobium*, *Pseudomonas*, *Rhodanobacter*, *Mycobacterium*, *Sphingomonas*, *Devosia*, *Rhodoplanes*, and *Flavobacterium* (Supplementary Figure S4). Additionally, these identified core bacterial microbiome taxa were also present at high relative abundances in the 3-year-old plants from the field nursery (Supplementary Figure S4).

### Relative Abundance of Functional Genes Related to N Cycling in Rhizosphere Samples *A. nordmanniana*

**FIGURE 5:**
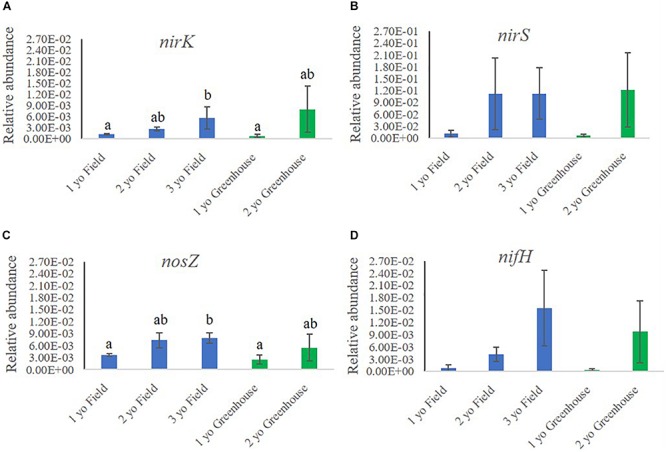
Relative abundance of functional genes involved in nitrogen cycling **(A)** *nirK*, **(B)** *nirS*, **(C)** *nosZ* and **(D)** *nifH*, in the rhizosphere samples of 1-, 2-, and 3-year-old (yo) plants collected in the field and greenhouse nurseries. The relative abundance of nitrogen cycling genes was calculated by the ratio of the absolute copy numbers of the nitrogen cycling genes of interest to the 16S rRNA gene. The figure shows average relative abundances of the target genes in each sample. Bars indicate standard error, *n* = 5.

We investigated the relative abundances of functional genes related to N cycling in rhizosphere samples collected in the field and greenhouse nurseries (Figure 5). Analysis of the functional genes involved in N cycling included *nirK* and *nirS* (nitrite reductases) and *nosZ* (nitrous-oxide reductase), all involved in denitrification. Further, *nifH* (nitrogenase iron protein) involved in N fixation and *amoA* (ammonia monooxygenase) involved in nitrification were also quantified.

In the field nursery, the relative abundance of *nirK*, *nirS*, and *nosZ* all increased with plant age, either significantly or by trend (Figure 5). Thus, the relative abundance of *nirK* was significantly higher (*P* < 0.05) in rhizosphere samples from 3-year-old than 1-year-old plants (Figure 5A). In the greenhouse nursery, the same tendency was noted, but differences were not significant (Figure 5A). Similarly, the relative abundance of *nirS* was higher in the older plants, particularly in the greenhouse nursery; however, there were no significant differences (Figure 5B). The relative abundance of *nosZ* followed the same pattern as noted for *nirK* and *nirS*, as it was higher (*P* < 0.05) in the rhizosphere of 3-year-old plants compared to 1-year-old plants in the field nursery (Figure 5C). Moreover, *nifH* also increased with plant age by trend in both the field and the greenhouse nurseries (Figure 5D), whereas the *amoA* copy numbers in all analyzed samples were below the detection threshold (data not shown).

### Transplant Experiment: *A. nordmanniana* Bacterial Community Diversity and Composition

**FIGURE 6:**
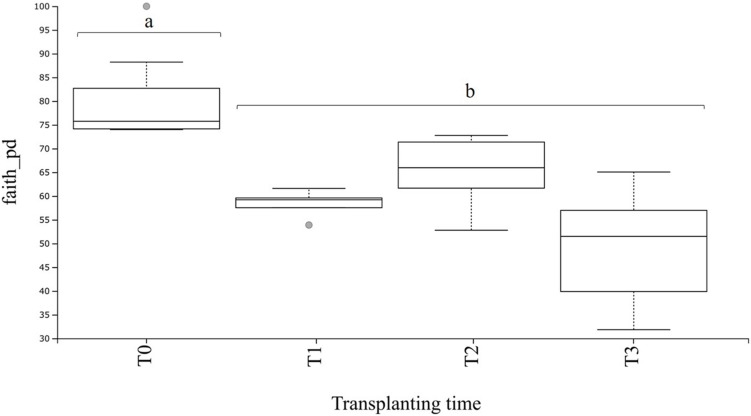
Boxplot of bacterial alpha diversity in *A. nordmanniana* rhizosphere samples. Analysis includes samples of 2-year-old plants from the greenhouse before (T0) and after transplanting to the field (T1 = 3 months after transplanting, T2 = 6 months after transplanting, and T3 = 9 months after transplanting). Alpha diversity was measured by Faith’s phylogenetic diversity index (faith_pd). Different small letters a, b represent significant differences with the corrected *P*-value (*q*-value < 0.05), after Kruskal–Wallis pairwise comparisons; gray dots indicate samples that represent outliers and each error bar represent the SD, *n* = 5.

From the dataset used for bacterial community analysis in the transplant experiment (*n* = 20 samples), a total of 9,025 ASVs were obtained. These ASVs appeared a total of 1,876,702 times among all samples (after quality filtering and trimming). As seen for the field and greenhouse samples, a plateau was also observable at a sequencing depth of 15,000 reads in the rarefaction curves (Supplementary Figure S5). The phylogenetic diversity of the rhizosphere communities at T0 (before transplanting) was higher (*P* < 0.05) as compared with values for the transplanted plants (Figure 6). Furthermore, there were no significant differences in the phylogenetic diversity between transplanted plants at T1 and T2, and T1 and T3, whereas a significant (*P* < 0.01) difference was observed between transplanted plants at T2 and T3 (Supplementary Table S6), whereas the corrected *q*-value showed no significant differences in the phylogenetic diversity between transplanted plants at T1, T2, and T3 (Figure 6 and Supplementary Table S6).

**FIGURE 7:**
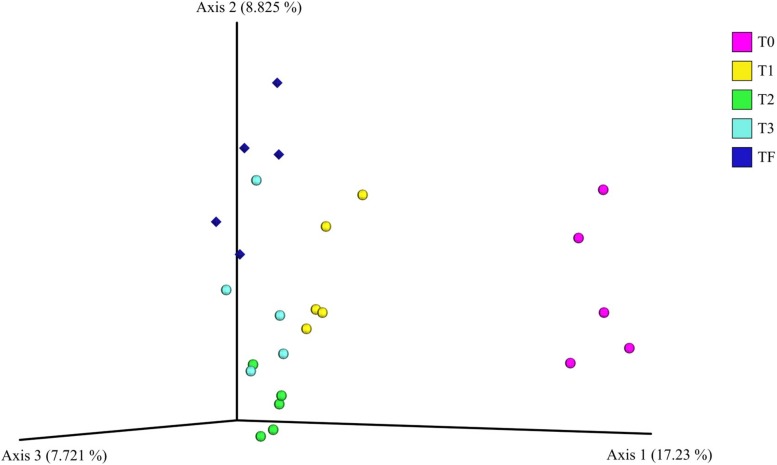
Principal coordinate analysis plot of the bacterial communities in the *A. nordmanniana* rhizosphere. The analysis included 2-year-old plants from field (TF), and 2-year-old plants included in the transplant experiment of greenhouse plants to the field: T0 = before transplanting, T1 = 3 months after transplanting, T2 = 6 months after transplanting, and T3 = 9 months after transplanting. Analysis is based on the unweighted-UNIFRAC distances. Diamonds are field samples of 2-year-old non-transplanted plants, and circles are greenhouse samples before and after transplanting to field nursery.

For the beta diversity analysis, we performed a comparison between the bacterial communities in the rhizospheres of the plants from the transplant experiment (T0, T1, T2, and T3). We even included the bacterial communities in the rhizosphere of 2-year-old plants from field that were sampled previously (TF). The PCoA plots showed that rhizosphere bacterial communities from the 2-year-old plants in the greenhouse before transplanting (T0) were dissimilar to the bacterial communities from the 2-year-old plants transplanted to field, that is, T1, T2, and T3 (Figure 7). Additionally, the communities from transplanted plants (T1, T2, and T3) with time became increasingly similar to the rhizosphere bacterial communities from 2-year-old plants grown in the field (TF) (Figure 7). Furthermore, a PERMANOVA indicated a significant (*P* < 0.001) difference across all samples, the individual *P*-values after the pairwise PERMANOVA are found in Supplementary Table S7.

In the transplant experiment, several bacterial phyla changed their relative abundances in rhizosphere with time after the transplanting. For example, Actinobacteria and TM7 both showed significant (*P* < 0.01) increases in their relative abundance in the transplanted plants at T3 compared with plants before transplanting (T0) (Supplementary Figure S6). In contrast, the relative abundance of Verrucomicrobia was lower (*P* < 0.05) at T3 than at T0, and a comparable change (*P* < 0.05) was recorded for Bacteroidetes, although with higher variability with time, and for Proteobacteria where the decrease was not significant (Supplementary Figure S6).

**FIGURE 8:**
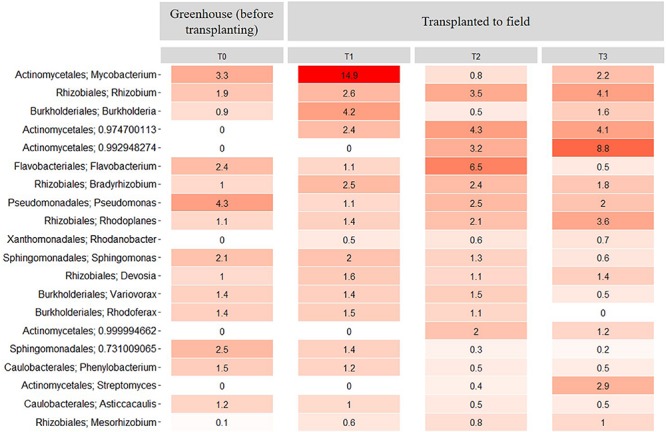
Heatmap showing the relative abundance of the 20 most abundant rhizosphere bacterial taxa at the genus level across all samples from the transplantation experiment. T0 = greenhouse plants before transplanting to field, T1 = 3 months after transplanting, T2 = 6 months after transplanting and T3 = 9 months after transplanting. Numbers represent the bacterial relative abundance percentages, and the darker shades of red in the figure represent the higher percentages of relative abundance at the bacterial genus level, whereas the lighter colors represent the lowest relative abundance at bacterial genus level per sample.

At the genus level, the relative abundance of *Mycobacterium* presented a significant (*P* < 0.01) increase between the rhizosphere samples at T0 and T1. However, at T2 and T3, the relative abundance of this genus decreased significantly (*P* < 0.01) when compared to the relative abundance at T1 (Figure 8). Further, the relative abundance of *Rhizobium* increased in the transplanted plants compared with the non-transplanted plants from 1.9% to 2.6%, 3.5%, and 4.1% at T1, T2, and T3, respectively (Figure 8). In contrast, the relative abundance of *Burkholderia* decreased significantly (*P* < 0.01) from 4.2% in the transplanted plants at T1 to 0.5 and 1.6% at T2 and T3, respectively (Figure 8). Additionally, some genera of the Actinomycetales as well as the genus *Rhodanobacter* were only observed in the rhizosphere samples from transplanted plants, and their relative abundance increased with time. In contrast, the relative abundance of *Pseudomonas* decreased (*P* < 0.05) after transplanting compared with the non-transplanted plants (Figure 8). Importantly, all the genera of the core microbiome, as presented above, were maintained among the 20 most abundant rhizosphere genera in the transplant experiment (Figure 8).

## Discussion

### The Interaction Between Plant Development and Production System Influences *A. nordmanniana* Bacterial Communities

It has been speculated that the interactions between tree hosts and their associated microbiota are more durable than those occurring in annual plants and that rhizosphere communities in perennial crops are primarily shaped by persistent changes such as environmental conditions (Mercado-Blanco et al., 2018). We therefore compared the effect of plant age and of production system on the rhizosphere bacterial communities of *A. nordmanniana.*

In the field nursery, the rhizosphere communities showed lower alpha diversity than the bulk soil communities as also seen for other tree species (Qin et al., 2019), and the most abundant bacterial phyla (Actinobacteria, Bacteroidetes, and Proteobacteria) were consistent with previous findings for the bacterial phyla associated with conifers (Yu et al., 2013; Shi et al., 2014; Naumova et al., 2015; Uroz et al., 2016; Proença et al., 2017), including *A. nordmanniana* (Garcia-Lemos et al., 2019).

For the greenhouse samples, several taxa were enriched in rhizosphere as compared to the growth medium. This was in particular the case for different genera belonging to Enterobacteriales, which was almost exclusively found in the greenhouse rhizosphere samples. Enterobacteriales are Gram-negative bacteria, which have been reported to be good degraders of organic compounds (Wüst et al., 2011; Cernava et al., 2019). Cernava et al. (2019) suggested that soils with high organic content might act as soil reservoirs from where bacterial members of Enterobacteriales can spread to the rhizosphere and colonize plant tissues facilitated by their high tolerance to a wide range of plant-specific metabolites (Cernava et al., 2019). In consequence, our results agree with previous studies reporting this order associated with high content of organic matter and in association with plants.

Several rhizosphere taxa showed dynamics with plant age in the field and greenhouse samples. In field samples, the relative abundance of *Burkholderia*, *Rhizobium*, *Bradyrhizobium*, *Sphingomonas*, and *Devosia* was dynamic across plant age. In the greenhouse, overall higher relative abundance of members of Enterobacteriales in the 1-year-old plants could indicate that the younger *A. nordmanniana* plants are more susceptible to colonization by this bacterial group. Moreover, the relative abundance of *Rhizobium*, *Bradyrhizobium*, *Rhodoplanes*, and *Pseudomonas* increased with plant age, whereas the relative abundance of *Burkholderia* decreased. This suggests that *A. nordmanniana* plants can recruit different groups of bacteria at different growth stages (Lareen et al., 2016). While information about temporal dynamics of rhizosphere microbiota is not available for conifers, it has been reported for annual crop plants that many of the highly abundant rhizosphere bacteria show more distinct temporal dynamics in the rhizosphere than in the bulk soil (Yang et al., 2017), suggesting a strong impact of the rhizosphere on the associated microorganisms. Root exudates play an important role in shaping the rhizosphere microbial communities (Chaparro et al., 2014), and the root exudate patterns in different plant species (Bais et al., 2006; Chaparro et al., 2014; Na et al., 2019), including *A. nordmanniana* (Rasmussen et al., 2009), can be altered with plant age. The temporal dynamics of bacterial taxa in the rhizosphere is important for the plant, as the rhizosphere has been described as a microbial “hot spot,” where the microorganisms actively interact with plant metabolic processes and have an important role for plant growth and health (Dennis et al., 2010).

We even observed significant effects of the production system on the bacterial communities associated to *A. nordmanniana*. The difference in bacterial communities between the field and the greenhouse nurseries was evident through differences in the relative abundance of dominating phyla and genera. Hence, *Rhizobium*, *Bradyrhizobium*, and *Devosia* had higher relative abundance in the field samples, whereas *Pseudomonas* and genera from the Enterobacteriales had higher abundances in the greenhouse samples. Even different temporal dynamics of major genera as *Rhizobium*, *Bradyrhizobium*, *Rhodoplanes*, *Pseudomonas*, and *Burkholderia* differed between field and greenhouse samples, indicating that the effect of plant age on the bacterial community composition is related with the conditions in the production system.

We could observe clear differences between bacterial communities in the soil and in the peat-based growth medium, and the soil characteristics were different between the field soil and the growth medium. In particular, the growth medium had a higher amount of organic matter and a lower pH than the field soil. Bacteroidetes was more abundant in the growth medium than in the bulk soil samples. Bacteroidetes is considered a copiotrophic phylum and is abundant in forest soils (Karimi et al., 2019) and in Arctic peatlands, where it shows metabolic potential for degradation of soil organic carbon (Tveit et al., 2013). Hence, the high amount of organic matter in the growth medium of the greenhouse nursery could influence the abundance of this phylum.

In the current study, we found a higher relative abundance of Acidobacteria belonging to subdivision 1 in the field soil than in the growth medium despite the higher pH of the soil. Soil pH is recognized as an important factor shaping soil bacterial communities (Rousk et al., 2010), but Acidobacteria subdivision 1 generally dominates at low pH (Kielak et al., 2016). However, the abundance of Acidobacteria is generally higher in soil with low resource availability, and Acidobacteria subdivision 1 correlates negatively with soil N, which was higher in the growth medium. Hence, the abundance of Acidobacteria seemed more affected by organic matter and N availability than of pH for the current bulk soil–growth medium comparison. Nevertheless, the effect of soil properties versus other physical and chemical factors (e.g., light, temperature, and precipitation) that vary between field and greenhouse nurseries cannot be disentangled in our current field study.

### Bacterial N-Cycling Potential Is Dynamic Across Plant Age

In the current study, we observed that the bacterial N-cycling potential in the rhizosphere of *A. nordmanniana* could be related to plant age, but not to the production system. For the *nirS*, *nirK*, and *nosZ* genes involved in denitrification, the dynamics of several taxa might contribute to the general increase in their relative abundance with plant age. For example, *Burkholderia*, which increased in their relative abundance by plant age in the field samples, has been reported to harbor copies of these denitrification genes (Yoshida et al., 2010; Saarenheimo et al., 2015; Espenberg et al., 2018; Meng et al., 2018). Moreover, the increase in *nirK*, and by trend *nirS*, might be correlated with the increase in the relative abundance of Acidobacteriales by plant age in the field nursery. Recent studies have shown that some Acidobacteria species harbors copies of *nirK* and *nirS* (Eichorst et al., 2018), although the involvement of Acidobacteria in the N-cycling processes such as N fixation, nitrification, or denitrification is still not clear (Kielak et al., 2016). However, the increases in denitrification genes in the greenhouse nursery could not be related to dynamics in the above taxa. It might be related to the increase with plant age in the relative abundance of *Pseudomonas* and *Sphingomonas*, as these bacterial genera contain members with denitrification characteristics (Deng et al., 2014; Hester et al., 2018; Li et al., 2018). It has been reported before that the C:N ratio influences denitrification process, as the abundance of denitrifiers positively responds to increased C availability in the soil environment (Miller et al., 2008, 2009; Henderson et al., 2010). Thus, a C-rich environment in the rhizosphere samples could lead to the increased abundance of denitrification-related genes in this environment. Furthermore, the high potential for denitrification could be partly explained by an anoxic microenvironment near the roots caused by the oxygen consumption of metabolically active microorganisms. The potential for N fixation also increased with plant age. The increase in relative abundance of *nifH* in the field nurseries coincided with a decrease in distinct members of Rhizobiales such as *Rhizobium*. However, not all Rhizobia harbor the *nifH* gene, and the rhizosphere can include non-fixing members of *Rhizobium* (Dicenzo et al., 2019). Alternatively, the increase in the *Burkholderia*, which includes N-fixing taxa, might be responsible for the observed increase in *nifH* in the field samples (Rodrigues Coelho et al., 2008; Wang et al., 2013). For the greenhouse samples, the trend for an increase in relative abundance of *nifH* coincided with an increase in *Rhizobium*, underlining that many different community structures may lead to increased potential for N fixation (Igiehon and Babalola, 2018). Overall, the observed increased in the relative abundance of *nifH* with age might be correlated with an increase in the C:N ratio caused by root exudates that could recruit N fixers to the rhizosphere (Bürgman et al., 2005).

N is an essential nutrient for tree growth, and N remobilization from soil to roots becomes more significant as the tree grows. This is because with age the potential storage of N increases, while the rate of N uptake slows (Millard and Grelet, 2010). Christmas trees growing on sandy soils with less than 3% of organic matter are typically in more need of N fertilization than trees growing on loamy soils (Heckman and Vodak, 2012). Therefore, the recruitment of rhizosphere bacteria involved in the N cycling, as well as the establishment of beneficial rhizosphere microbial associations, is crucial for the development of the Christmas trees, particularly in nutrient-poor sandy soils. For instance, it has been proposed that the presence of N fixing bacteria in the rhizosphere of conifers may explain the ability these plants to grow under N limitation (Mercado-Blanco et al., 2018).

### The Rhizosphere Core Bacterial Community of *A. nordmanniana* Is Maintained Across Nursery Production System and Plant Age

We determined that the *A. nordmanniana* core bacterial rhizosphere community is maintained across productions system and plant age. This core microbiome is composed primarily by *Burkholderia*, *Rhizobium*, *Bradyrhizobium*, *Pseudomonas*, *Rhodanobacter*, *Mycobacterium*, *Sphingomonas*, *Devosia*, *Rhodoplanes*, and *Flavobacterium.* The current results agree with observations previously reported for the *A. nordmanniana* rhizosphere core bacterial microbiome as analyzed at the order level for 3-year-old plants coming from different field locations (Garcia-Lemos et al., 2019).

The rhizosphere core bacterial microbiome of *A. nordmanniana* includes reported plant beneficial genera such as *Burkholderia* and *Rhizobium*. These genera harbor N-fixing and mineral-weathering bacteria (Sun et al., 2014; Garrido-Oter et al., 2018) and are part of the core microbiomes for a wide range of plant hosts (Yeoh et al., 2017; Garrido-Oter et al., 2018). Additionally, in our previous study of 3-year-old *A. nordmanniana* plants, we reported that members of Agaricales were predominant in the fungal core microbiome (Garcia-Lemos et al., 2019). The order Agaricales includes many EM species (Tedersoo and Smith, 2013), which form associations with bacteria as non-nodulating *Rhizobium* and some members of *Burkholderia*, *Xanthomonas*, and *Pseudomonas* at the root tips of many conifer species (Khetmalas et al., 2002; Izumi et al., 2006; Nguyen and Bruns, 2015; Lladó et al., 2017). Hence, the high abundance of these bacterial core genera associated with roots of *A. nordmanniana* could be influenced by their interactions with EM (Nguyen and Bruns, 2015).

### The Core Bacterial Community Is Stable During Transplantation of the Plants From Greenhouse Nursery Conditions to the Field

The transplant experiment showed that bacterial communities of the plants transplanted from the greenhouse of the field became increasingly similar to communities from field plants of the same age. Nevertheless, *A. nordmanniana* was able to maintain its rhizosphere core microbiome. Currently, there are few studies reporting the effect of transplantation on root-associated microbial communities. However, Edwards et al. (2018) reported that the root microbiota of 2-week-old rice transplanted from the greenhouse to the field shifted to become more similar to those hosted by rice plants in the field. The same authors reported that the major shift in the composition of the root microbiota was correlated with the plant transition to reproductive growth and that distinct root microbiota occurred for the juvenile and adult plant phases (Edwards et al., 2018).

In Christmas trees grown in Denmark (such as *A. nordmanniana*), the root development is strong during the mid-late summer (July–September), and according to the growers, planting or transplanting should be carried out during that period (S. Sørensen, personal communication, July, 2017)^6^. Our transplant experiment was initiated in August, and therefore our results provide a realistic example of the composition, establishment, and dynamics of rhizosphere bacterial communities in the transplanted plants. A correct and fast early seedling development is critical for survival and establishment of Christmas trees after the nursery stage (Seifert, 2015), and the Christmas tree establishment phase begins the first year following (trans)planting. Knowledge on the dynamics and stability of rhizosphere bacterial communities during early seedling growth and transplanting is therefore a requirement for developing microbiological inoculants for improved plant development, which can be applied to the seedlings, or added during transplanting, and be stably maintained at the roots during tree establishment.

## Conclusion

Referring back to the research questions we posed in the *Introduction*, our findings can be summarized as follows: (1) the relative abundance of bacterial taxa as well as the N-cycling potential of rhizosphere bacterial communities is dynamic across plant age. (2) However, our findings suggest that the nursery production systems also strongly influence the bacterial dynamics. Nevertheless, the effect of soil type versus nursery management in our study cannot be disentangled. Therefore, future studies analyzing these two factors individually will be needed to uncover their distinct impacts on the *A. nordmanniana* bacterial community composition. (3) *A. nordmanniana* plants maintain a core rhizosphere microbiome across plant age and nursery production systems. The core microbiome is mostly composed of potentially beneficial bacterial, such as N fixers, and plant growth promoters. (4) The core microbiome is even maintained during the transplantation of plants from the greenhouse to the field. Future studies are required to provide detailed knowledge about the possible mechanisms that different microbial groups employ during association with *A. nordmanniana*, for nutrient turnover in soil and rhizosphere. Such studies should focus on relations between the absolute abundance of taxa, within the core microbiome and the plant nutrient concentrations, and the activities of enzymes involved in rhizosphere soil nutrient transformations. Additionally, it would be important to mine the rhizosphere bacterial communities by cultivation procedures to recover beneficial microbes that can be used for growth promotion of *A. nordmanniana* in field and greenhouse nurseries. Several soil-borne fungal and oomycete pathogens (e.g., from the genera *Fusarium*, *Pythium*, and *Rhizoctonia*) may cause *A. nordmanniana* seedling loss in nurseries. On the other side, several EM fungi support plant health, and ectomycorrhization in *Abies* species has been previously reported. Hence, it will be important to map their distribution in the field and greenhouse production systems and to investigate their interactions with members of the bacterial communities.

## Data Availability Statement

The datasets generated for this study can be found in the PRJNA515250.

## Author Contributions

AG-L: experimental design, sample collection and processing, acquisition and analysis of the data and interpretation, and manuscript writing. AG: processing of the raw sequences on QIIME platform, bioinformatics analysis, results generation and interpretation, and manuscript writing. MN: contributions to experimental design and data analysis and manuscript writing. LH and TR: contribution to data analysis and revising the manuscript. BV: experimental design, sampling, and revising the manuscript. ON: experimental design, sampling, analysis of the data and interpretation, and manuscript writing.

## Conflict of Interest

The authors declare that the research was conducted in the absence of any commercial or financial relationships that could be construed as a potential conflict of interest.

## Footnotes

1 https://www.hdnordictrees.com/

2 http://www.himmerlands.com

3 www.eurofins.dk

4 https://github.com/qiime2/q2-feature-classifier

5 www.jiffygroup.com

6 Sørensen, S. (2017). *Personal Interview with Veierskov, B*.
