# Supplementary material for: Under the Christmas Tree: Belowground Bacterial Associations With Abies nordmanniana Across Production Systems and Plant Development
Source: Front Microbiol. 2020 Mar 4;11:198. doi: 10.3389/fmicb.2020.00198 (PMC7064441; doi:10.3389/fmicb.2020.00198)
Supplement: Supplementary file 1 [file Data_Sheet_1.PDF]

Supplementary material

Supplementary Table S1. Primers used for qPCR assays

**Target genes**	**Primers**	**Sequences**	**Annealing Temperature**	**References**
16S rDNA	341F<br>805R	CCTAYGGGRBGCASCAG<br>GGACTACNNGGGTATCTAAT	58˚C	(Yu et al., 2005)
nirS-1	nirS cd3AF<br>nirS R3cd	GTSAACGTSAAGGARACSGG<br>GASTTCGGRTGSGTCTTGA	56˚C	(Zheng et al., 2018)
nifH	nifHF<br>nifHRb	AAAGGYGGWATCGGYAARTCCACCAC<br>TGSGCYTTGTCYTCRCGGATBGGCAT	56˚C	(Zheng et al., 2018)
nirK	nirK-F1aCu<br>nirK-R3Cu	ATCATGGTSCTGCCGCG<br>GCCTCGATCAGRTTGTGGTT	58˚C	(Zheng et al., 2018)
nosZ	nosZ-F<br>nosZ-1622R	CGYTGTTCMTCGACAGCCAG<br>CGSACCTTSTTGCCSTYGCG	63˚C	(Kloos et al., 2001)
amoA	amoA-1Fmod<br>GenAOBR-1	CTGGGGTTTCTACTGGTGGTC<br>GCAGTGATCATCCAGTTGCG	58˚C	(Meinhardt et al., 2015)

References

Kloos, K., Mergel, A., Rösch, C., & Bothe, H. (2001). Denitrification within the genus Azospirillum and other associative bacteria. *Functional Plant Biology*, *28*, 991-998. https://doi.org/10.1071/PP01071

Meinhardt, K. A., Bertagnolli, A., Pannu, M. W., Strand, S. E., Brown, S. L., & Stahl, D. A. (2015). Evaluation of revised polymerase chain reaction primers for more inclusive quantification of ammonia-oxidizing archaea and bacteria. *Environmental Microbiology Reports*, *7*, 354–363. https://doi.org/10.1111/1758-2229.12259

Yu, Y., Lee, C., Kim, J., & Hwang, S. (2005). Group-specific primer and probe sets to detect methanogenic communities using quantitative real-time polymerase chain reaction. *Biotechnology and Bioengineering*, *89*, 670–679. https://doi.org/10.1002/bit.20347

Zheng, B., Zhu, Y., Sardans, J., Peñuelas, J., & Su, Q. (2018). QMEC : A tool for high-throughput quantitative assessment of microbial functional potential in C, N, P, and S biogeochemical cycling. Sci. China Life Sci. 61, 1451–1462.

Supplementary Table S2. Bulk soil composition from sampling site in field nursery Primo Plant Ejendomme ApS, Denmark. Eurofins Agro Testing Denmark A/S (www.eurofins.dk) performed the soil analysis.

	Field (Primo Plant Ejendomme ApS)		
**Test (soil pack for Christmas tree)**	**1-year-old plants**	**2-year-old plants**	**3-year-old plants**
Sulfur in Sulphate-ICP-OES	<10 mg/kg DM.	<10 mg/kg DM.	<10 mg/kg DM.
Manganese (Mn)- ICP-OES	1.5 mg/kg DM.	1.2 mg/kg DM.	2.5 mg/kg DM.
Boron (B)- ICP-OES	0.1 mg/kg DM.	0.1mg/kg DM.	0.1 mg/kg DM.
Reaction Number-Potentiometry	5.9	5.9	5.5
Phosphorus (P)-Spectroscopy (FIA)	4.7 mg/100 g DM.	4.4 mg/100 g DM.	6.6 mg/100 g DM.
Potassium (K) (available for plant)-Spectroscopy (FIA)	9.3 mg/100 g DM.	8.5 mg/100 g DM	10.5 mg/100 g DM
Magnesium (Mg) (available for plant)-ICP-OES	6.3 mg/100 g DM.	7.1 mg/100 g DM.	6.2 mg/100 g DM.
Sodium (Na) (available for Plant)-ICP-OES	2.6 mg/100 g DM.	2.1 mg/100 g DM.	2.8 mg/100 g DM.
Copper (Cu)-ICP-OES	2.2 mg/kg DM.	2.3 mg/kg DM.	2.5 mg/kg DM.
Iron (Fe)- ICP-OES	90 mg/kg DM.	92 mg/kg DM.	138.5 mg/kg DM.
Zinc (Zn)-ICP-OES	1.8 mg/kg DM.	2 mg/kg DM.	2 mg/kg DM.
Nitrogen-ISO 13878 / Dumas method	0.2 % (w/w) DM.	0.2 % (w/w) DM.	0.2 % (w/w) DM.
Organic matter - ISO 11277	1.0%	1.2%	1%
pH	5.5 +/- 0.1	5.5 +/- 0.1	5.4 +/- 0.1

ICP-OES= Inductively Coupled Plasma – Optical Emission Spectrometry; FIA= Flow Injection Analysis- spectrometry; DM= Dry Matter.

Supplementary Table S3. Composition of the Jiffy® plant growth medium, from sampling site in greenhouse nursery; Himmerlands ApS, Denmark. Eurofins Agro Testing Denmark A/S (www.eurofins.dk) performed the soil analysis.

	Greenhouse (Himmerlands ApS)	
**Test (soil pack for Christmas tree)**	**1-year-old plants**	**2-year-old plants**
Manganese (Mn)- ICP-OES	22 mg/kg DM.	22 mg/kg DM.
Boron (B)- ICP-OES	5 mg/kg DM.	5 mg/kg DM.
Reaction Number-Potentiometry	5.8	5.8
Copper (Cu)-ICP-OES	2.2 mg/kg DM.	2.2 mg/kg DM.
Iron (Fe)- ICP-OES	140 mg/kg DM.	142 mg/kg DM.
Zinc (Zn)-ICP-OES	5.8 mg/kg DM.	6 mg/kg DM.
Nitrogen-ISO 13878 / Dumas method	0.6% (w/w) DM.	0.6% (w/w) DM.
Organic matter - ISO 11277	46.8%	47%
pH	4.5 +/- 0.1	4.5 +/- 0.1

ICP-OES= Inductively Coupled Plasma – Optical Emission Spectrometry; FIA= Flow Injection Analysis- spectrometry; DM= Dry Matter.

Supplementary Table S4. Results from pairwise of the differences between bacterial communities in the bulk soil from field, growth medium from greenhouse and rhizosphere from both sampling sites across all plant ages. Results based on the Kruskal-Wallis pairwise comparisons among all groups, n=5.

Group 1	Group 2	H	p-value	q-value
Field_bulk_soil_1_year	Field_bulk_soil_2_year	0.010909091	0.916814949	0.916814949
	Field_bulk_soil_3_year	1.32	0.250592051	0.341716433
	Field_rhizosphere_1_year	6.818181818	0.009023439	0.02900391
	Field_rhizosphere_2_year	6.818181818	0.009023439	0.02900391
	Field_rhizosphere_3_year	0.883636364	0.347207639	0.434009549
	Greenhouse_growth medium_1_year	5.770909091	0.016293604	0.040734009
	Greenhouse_growth medium_1_year	0.534545455	0.4647021	0.51003889
	Greenhouse_rhizosphere_1_year	1.32	0.250592051	0.341716433
	Greenhouse_rhizosphere_2_year	1.843636364	0.174525341	0.280487154
Field_bulk_soil_2_year	Field_bulk_soil_3_year	1.32	0.250592051	0.341716433
	Field_rhizosphere_1_year	6.818181818	0.009023439	0.02900391
	Field_rhizosphere_2_year	6.818181818	0.009023439	0.02900391
	Field_rhizosphere_3_year	0.534545455	0.4647021	0.51003889
	Greenhouse_growth medium_1_year	5.770909091	0.016293604	0.040734009
	Greenhouse_growth medium_2_year	0.272727273	0.601508134	0.644473001
	Greenhouse_rhizosphere_1_year	0.883636364	0.347207639	0.434009549
	Greenhouse_rhizosphere_2_year	1.32	0.250592051	0.341716433
Field_bulk_soil_3_year	Field_rhizosphere_1_year	6.818181818	0.009023439	0.02900391
	Field_rhizosphere_2_year	6.818181818	0.009023439	0.02900391
	Field_rhizosphere_3_year	3.152727273	0.075800175	0.142125327
	Greenhouse_growth medium_1_year	5.770909091	0.016293604	0.040734009
	Greenhouse_growth medium_2_year	2.454545455	0.117185087	0.195308479
	Greenhouse_rhizosphere_1_year	2.454545455	0.117185087	0.195308479
	Greenhouse_rhizosphere_2_year	3.938181818	0.047201768	0.09654907
Field_rhizosphere_1_year	Field_rhizosphere_2_year	2.454545455	0.117185087	0.195308479
	Field_rhizosphere_3_year	6.818181818	0.009023439	0.02900391
	Greenhouse_growth medium_1_year	3.152727273	0.075800175	0.142125327
	Greenhouse_growth medium_2_year	6.818181818	0.009023439	0.02900391
	Greenhouse_rhizosphere_1_year	4.810909091	0.028280123	0.063630276
	Greenhouse_rhizosphere_2_year	6.818181818	0.009023439	0.02900391
Field_rhizosphere_2_year	Field_rhizosphere_3_year	6.818181818	0.009023439	0.02900391
	Greenhouse_growth medium_1_year	6.818181818	0.009023439	0.02900391
	Greenhouse_growth medium_2_year	6.818181818	0.009023439	0.02900391
	Greenhouse_rhizosphere_1_year	6.818181818	0.009023439	0.02900391
	Greenhouse_rhizosphere_2_year	6.818181818	0.009023439	0.02900391
Field_rhizosphere_3_year	Greenhouse_growth medium_1_year	4.810909091	0.028280123	0.063630276
	Greenhouse_growth medium_2_year	0.010909091	0.916814949	0.916814949
	Greenhouse_rhizosphere_1_year	0.534545455	0.4647021	0.51003889
	Greenhouse_rhizosphere_2_year	1.32	0.250592051	0.341716433
Greenhouse_growth medium_1_year	Greenhouse_growth medium_2_year	5.770909091	0.016293604	0.040734009
	Greenhouse_rhizosphere_1_year	0.534545455	0.4647021	0.51003889
	Greenhouse_rhizosphere_2_year	3.938181818	0.047201768	0.09654907
Greenhouse_growth medium_2_year	Greenhouse_rhizosphere_1_year	0.534545455	0.4647021	0.51003889
	Greenhouse_rhizosphere_2_year	0.883636364	0.347207639	0.434009549
Greenhouse_rhizosphere _1_year	Greenhouse_rhizosphere_2_year	0.010909091	0.916814949	0.916814949

Supplementary Table S5. Pairwise PERMANOVA results of the differences between bacterial communities in the bulk soil from field, growth medium from greenhouse and rhizosphere from both sampling sites across all plant ages. Results based on the unweighted UniFrac distance matrix, n=10 and 999 permutations.

Group 1	Group 2	pseudo-F	p-value	q-value
	Field_bulk_soil_2_year	2.02431626	0.009	0.013235
	Field_bulk_soil_3_year	4.406106422	0.01	0.013235
	Field_rhizosphere_1_year	4.005029427	0.007	0.013235
	Field_rhizosphere_2_year	4.551105573	0.01	0.013235
	Field_rhizosphere_3_year	4.194017484	0.012	0.013846
	Greenhouse_growth_medium_1_year	6.438205602	0.014	0.015
	Greenhouse_ growth_medium _2_year	5.309455386	0.009	0.013235
	Greenhouse_rhizosphere_1_year	6.189567935	0.012	0.013846
Field_bulk_soil_1_year	Greenhouse_rhizosphere_2_year	4.903213592	0.007	0.013235
	Field_bulk_soil_3_year	2.586846543	0.01	0.013235
	Field_rhizosphere_1_year	3.708660866	0.01	0.013235
	Field_rhizosphere_2_year	3.472706197	0.006	0.013235
	Field_rhizosphere_3_year	2.878091701	0.009	0.013235
	Greenhouse_ growth_medium _1_year	5.168990674	0.008	0.013235
	Greenhouse_ growth_medium _2_year	4.512921316	0.008	0.013235
	Greenhouse_rhizosphere_1_year	5.258794714	0.006	0.013235
Field_bulk_soil_2_year	Greenhouse_rhizosphere_2_year	4.012907542	0.009	0.013235
	Field_rhizosphere_1_year	4.964039494	0.013	0.014268
	Field_rhizosphere_2_year	4.283818183	0.011	0.013846
	Field_rhizosphere_3_year	2.073276652	0.007	0.013235
	Greenhouse_ growth_medium _1_year	4.851252503	0.012	0.013846
	Greenhouse_ growth_medium _2_year	4.598431873	0.01	0.013235
	Greenhouse_rhizosphere_1_year	4.780122555	0.006	0.013235
Field_bulk_soil_3_year	Greenhouse_rhizosphere_2_year	3.970891688	0.008	0.013235
	Field_rhizosphere_2_year	1.826591036	0.008	0.013235
	Field_rhizosphere_3_year	3.491464512	0.004	0.013235
	Greenhouse_ growth_medium _1_year	5.004903811	0.016	0.016
	Greenhouse_ growth_medium _2_year	4.62477694	0.009	0.013235
	Greenhouse_rhizosphere_1_year	5.129768294	0.01	0.013235
Field_rhizosphere_1_year	Greenhouse_rhizosphere_2_year	3.571071357	0.004	0.013235
	Field_rhizosphere_3_year	2.641444652	0.008	0.013235
	Greenhouse_ growth_medium _1_year	4.31924371	0.013	0.014268
	Greenhouse_ growth_medium _2_year	4.533149098	0.015	0.015341
	Greenhouse_rhizosphere_1_year	4.076161549	0.006	0.013235
Field_rhizosphere_2_year	Greenhouse_rhizosphere_2_year	3.354279456	0.01	0.013235
	Greenhouse_ growth_medium _1_year	3.877767707	0.012	0.013846
	Greenhouse_ growth_medium _2_year	3.84050695	0.006	0.013235
	Greenhouse_rhizosphere_1_year	2.827641014	0.007	0.013235
Field_rhizosphere_3_year	Greenhouse_rhizosphere_2_year	2.69201795	0.009	0.013235
	Greenhouse_ growth_medium _2_year	4.224283213	0.015	0.015341
	Greenhouse_rhizosphere_1_year	3.935575634	0.01	0.013235
Greenhouse_ growth_medium _1_year	Greenhouse_rhizosphere_2_year	3.419941763	0.01	0.013235
	Greenhouse_rhizosphere_1_year	5.32933182	0.007	0.013235
Greenhouse_ growth_medium _2_year	Greenhouse_rhizosphere_2_year	1.851181536	0.01	0.013235
Greenhouse_rhizosphere _1_year	Greenhouse_rhizosphere_2_year	3.407267933	0.005	0.013235

Supplementary Table S6. Results from pairwise of the differences between rhizosphere bacterial communities of two-year-old, *Abies nordmanniana* from field (TF), and two-year-old plants included in the transplant experiment of greenhouse plants to the field: T0 = before transplanting, T1 = three months after transplanting, T2 = six months after transplanting and T3 = nine months after transplanting to field nursery in Denmark. Results based on the Kruskal-Wallis pairwise comparisons among all groups, n=5.

**Group 1**	**Group 2**	**H**	**p-value**	**q-value**
T0	T1	9.391771	0.00218	0.005449
	T2	9.391771	0.00218	0.005449
	T3	9.391771	0.00218	0.005449
T1	T2	2.454545	0.117185	0.146481
	T3	1.843636	0.174525	0.193917
T2	T3	3.938182	0.047202	0.067431

Supplementary Table S7. Pairwise PERMANOVA results of the differences between rhizosphere bacterial communities of two-year-old, *Abies nordmanniana* from field (TF), and two-year-old plants included in the transplant experiment of greenhouse plants to the field: T0 = before transplanting, T1 = three months after transplanting, T2 = six months after transplanting and T3 = nine months after transplanting to field nursery in Denmark. Results based on the unweighted UniFrac distance matrix, with 999 permutations.

**Group 1**	**Group 2**	**Sample size**	**pseudo-F**	**p-value**	**q-value**
	T1	15	3.728422857	0.003	0.0075
	T2	15	4.318621769	0.002	0.0066
	T3	15	4.377486055	0.001	0.005
T0	TF	15	4.96457933	0.001	0.005
	T2	10	2.614975465	0.005	0.01
	T3	10	2.638172521	0.009	0.01
T1	TF	10	2.721630585	0.016	0.016
	T3	10	1.93679566	0.009	0.01
T2	TF	10	2.616200778	0.007	0.01
T3	TF	10	1.958442373	0.007	0.01

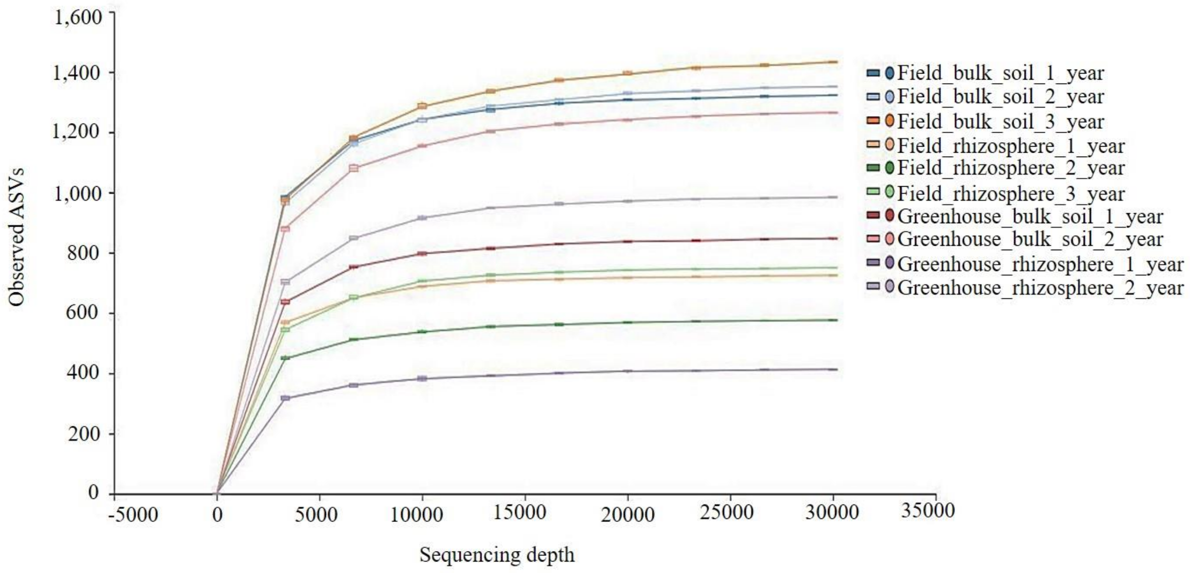

Supplementary Figure S1. Rarefraction curves, observed ASVs in the bulk soil (field samples), growth medium (greenhouse samples) and rhizosphere of one, two and three-year-old *A. nordmanniana* plants collected from field and greenhouse (one and two-year-old only) in Denmark. Each curve represent the average of five biological samples.

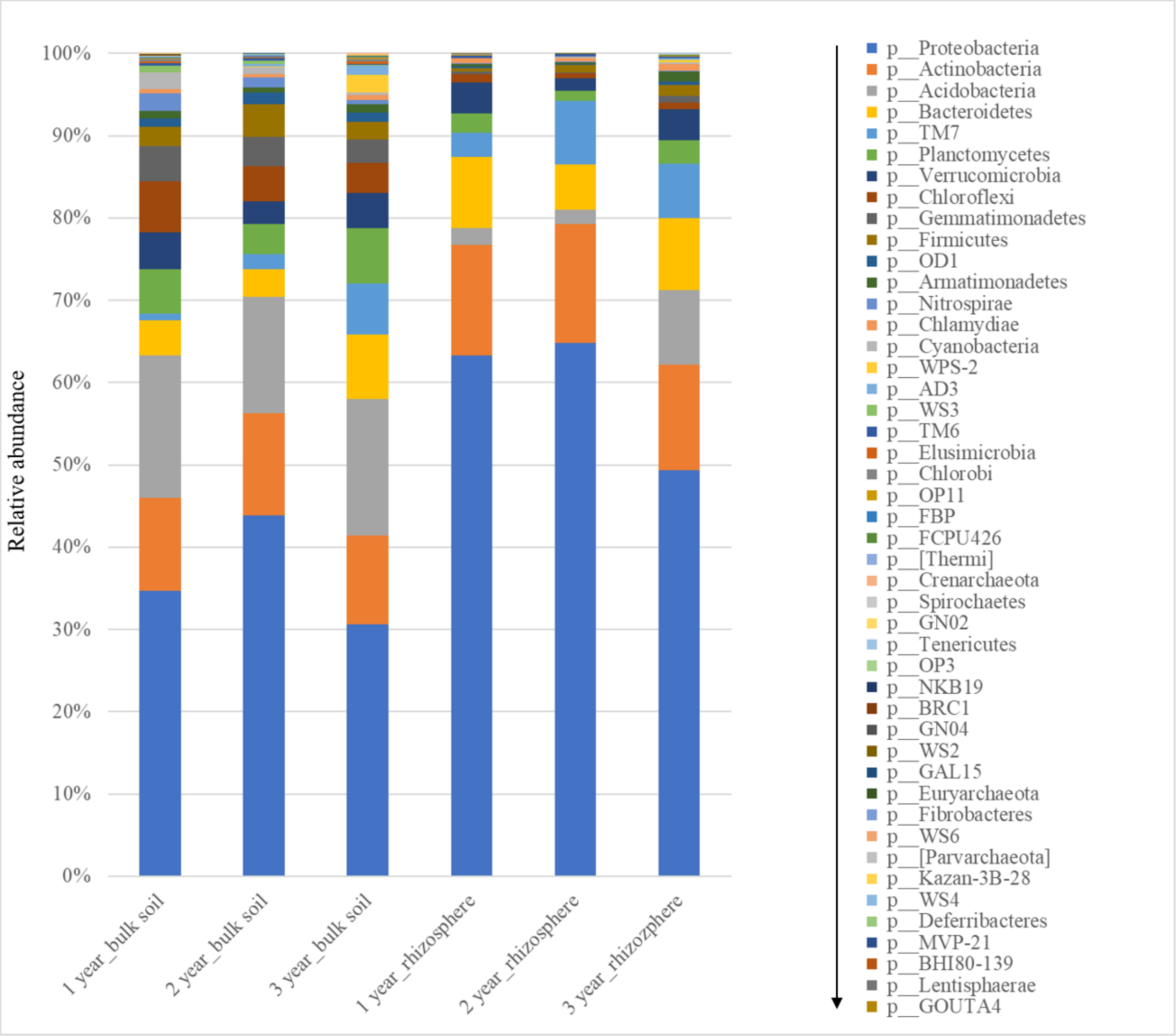

Supplementary Figure S2. Stacked bar plot showing relative abundances of bacterial phyla in the rhizosphere and bulk soil of one, two and three-year-old, *Abies nordmanniana* from field nursery in Denmark. Plot based on sequencing data for 16S rRNA genes. Phyla (P__) names are shown in descendent order (arrow), from the phylum with higher relative abundance to the phylum with the lowest relative abundance.

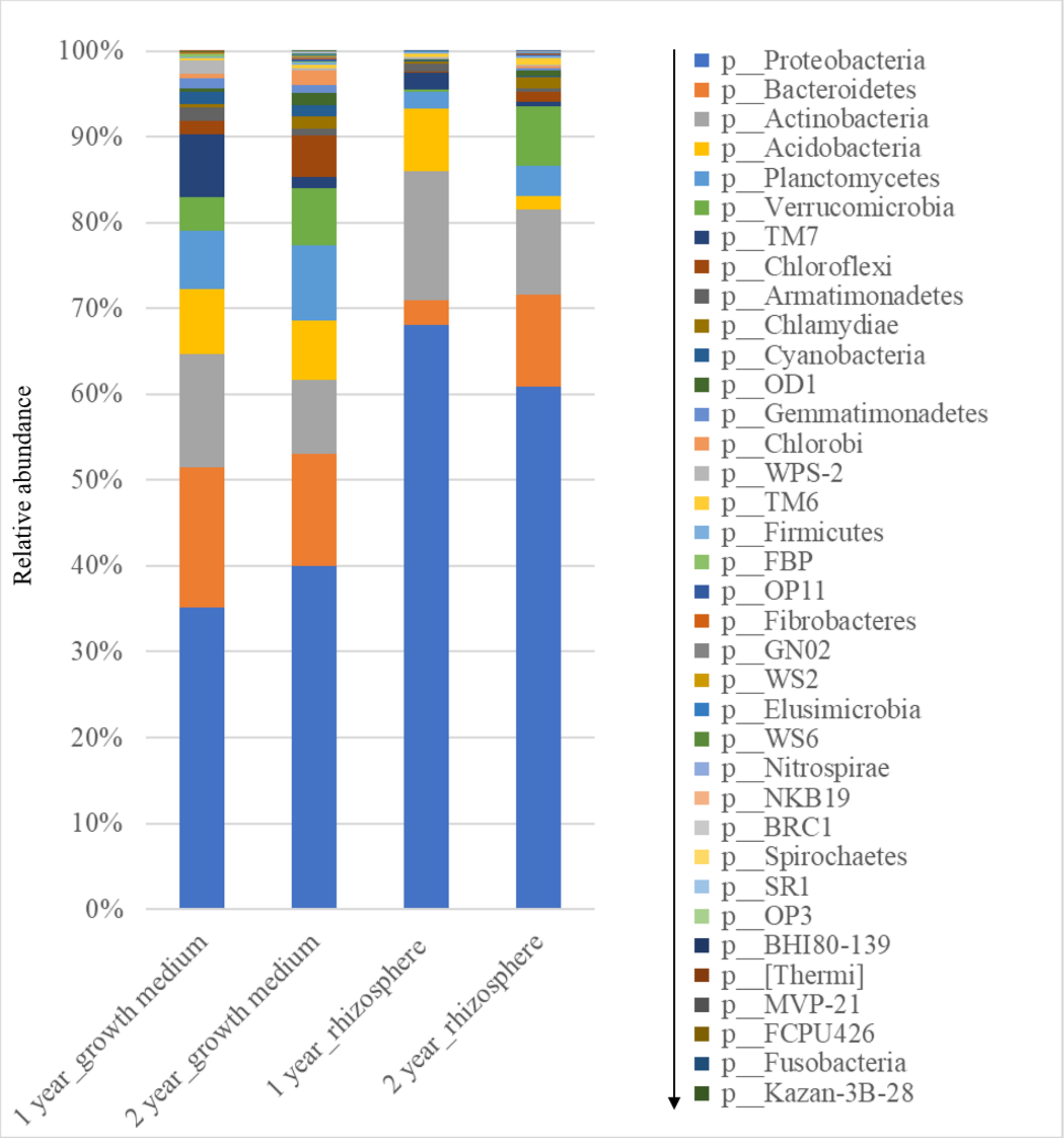

Supplementary Figure S3. Stacked bar plot showing relative abundances of bacterial phyla in the rhizosphere and peat-based growth medium of one, and two-year-old, *Abies nordmanniana* from greenhouse nursery in Denmark. Plot based on sequencing data for 16S rRNA genes. Phyla (P__) names are shown in descendent order (arrow), from the phylum with higher relative abundance to the phylum with the lowest relative abundance.

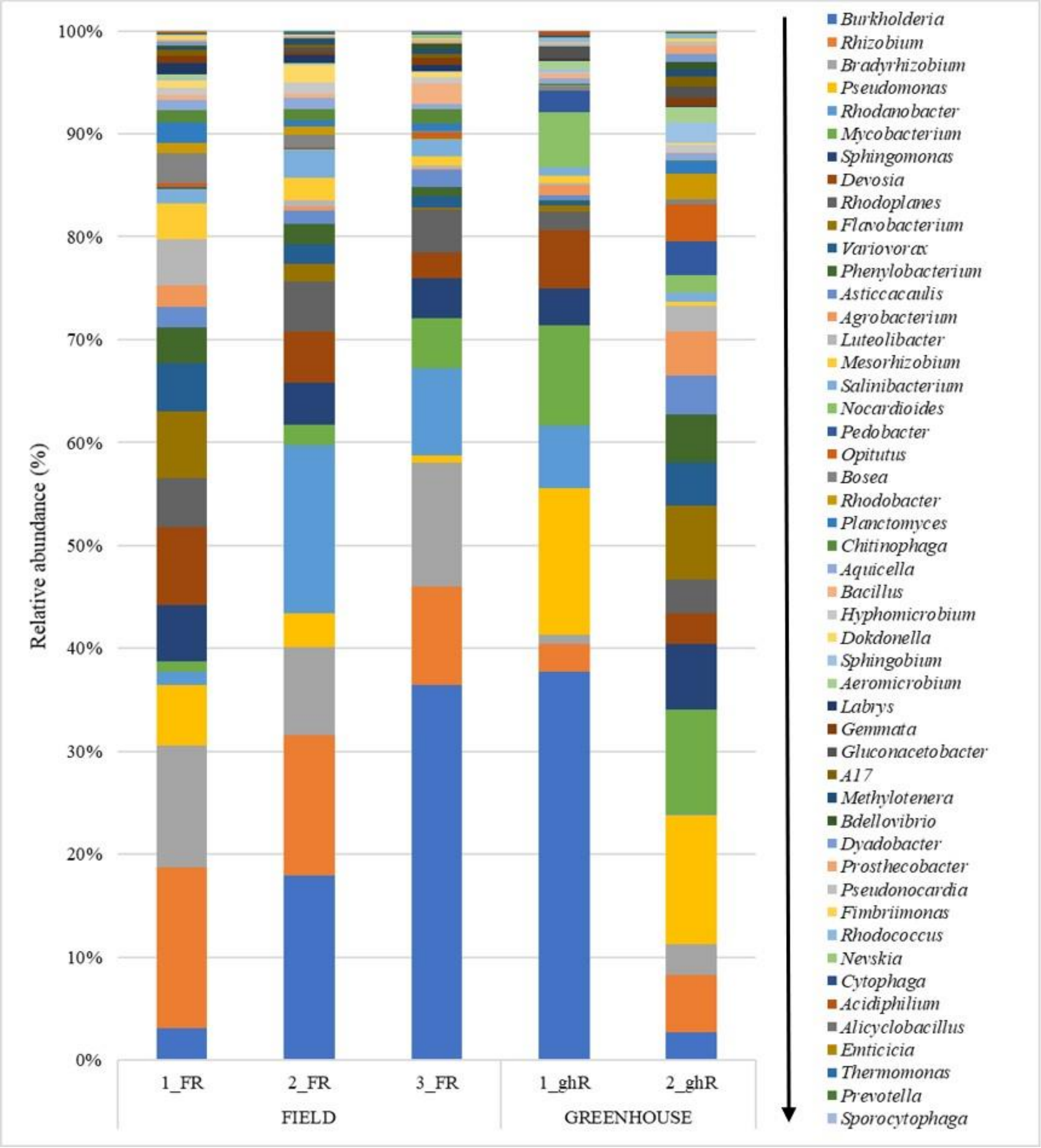

Supplementary Figure S4. Shared taxa at genus level in the rhizosphere of one, two and three-year old *A. nordmanniana* plants collected in field and greenhouse nursery in Denmark. Taxa names are shown in descendent order (arrow), from the genus with higher relative abundance to the genus with the lowest relative abundance.

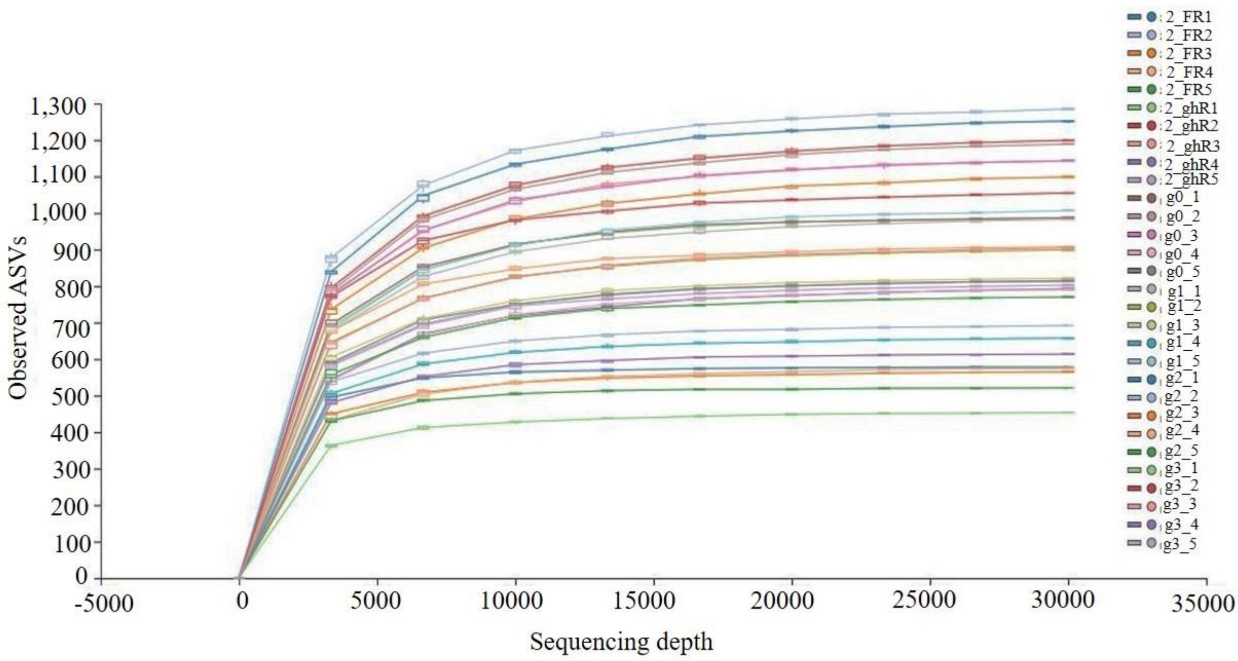

Supplementary Figure S5. Rarefraction curves, observed ASVs in the rhizosphere of two-year-old plants *A. nordmanniana* from field (FR), greenhouse (ghR) and before and after transplanted to field from greenhouse (g0, g1, g2 and g3).

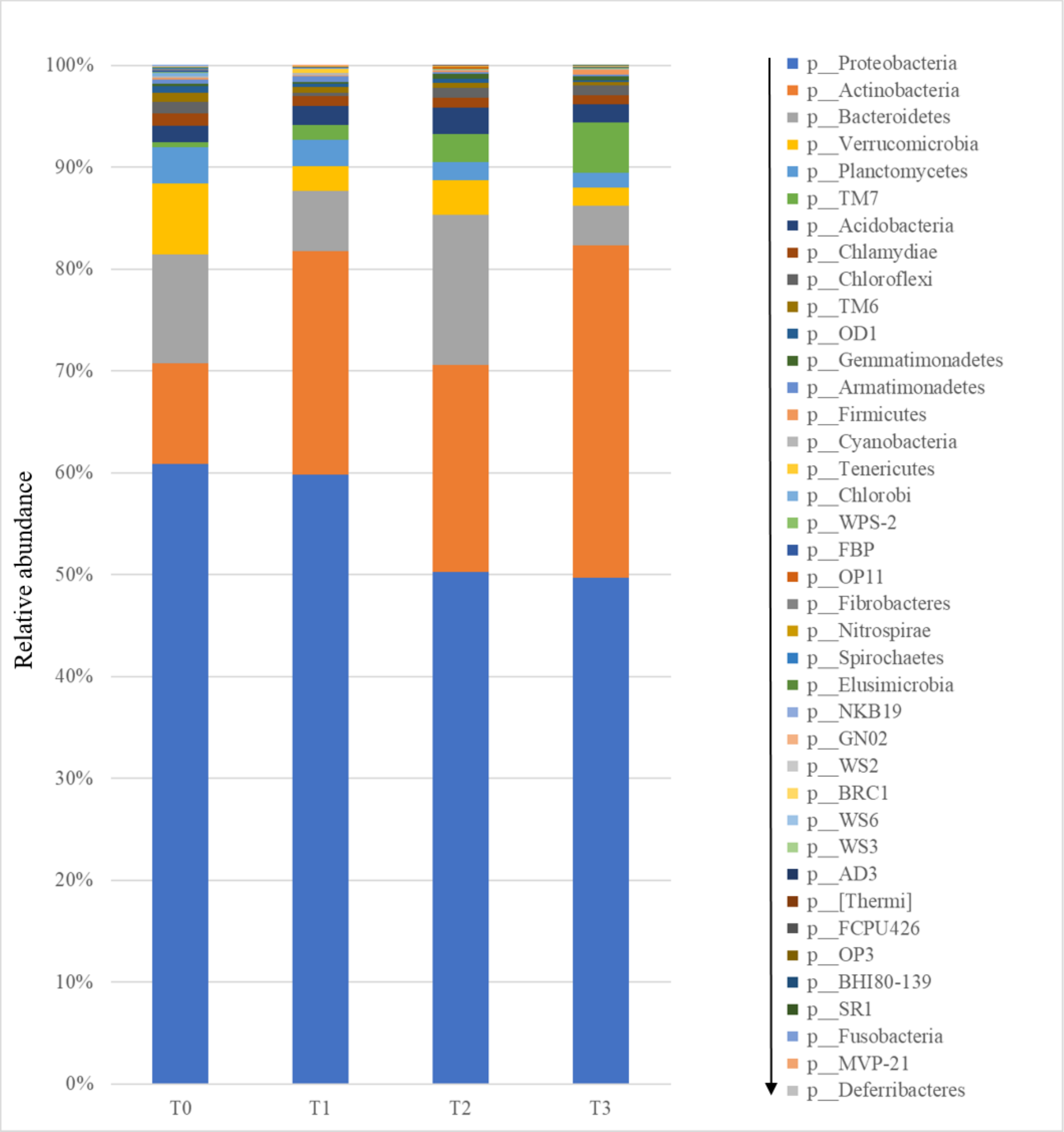

Supplementary Figure S6. Stacked bar plot showing relative abundances of bacterial phyla in the rhizosphere of two-year-old, *Abies nordmanniana* plants from the transplant experiment. Greenhouse plants before transplanted (T0) and after transplanted at T1 (three months), T2 (six months) and T3 (nine months) of transplanting to field nursery in Denmark. Plot based on sequencing data for 16S rRNA genes. Phyla (P__) names are shown in descendent order (arrow), from the phylum with higher relative abundance to the phylum with the lowest relative abundance.
